# Supplementary material for: Human kidney is a target for novel severe acute respiratory syndrome coronavirus 2 infection
Source: Nat Commun. 2021 May 4;12:2506. doi: 10.1038/s41467-021-22781-1 (PMC8096808; doi:10.1038/s41467-021-22781-1)
Supplement: Supplementary file 1 — Supplementary Information [file 41467_2021_22781_MOESM1_ESM.pdf]

Supplementary Figure 1 The parameters of renal function in different groups of COVID-19 patients.

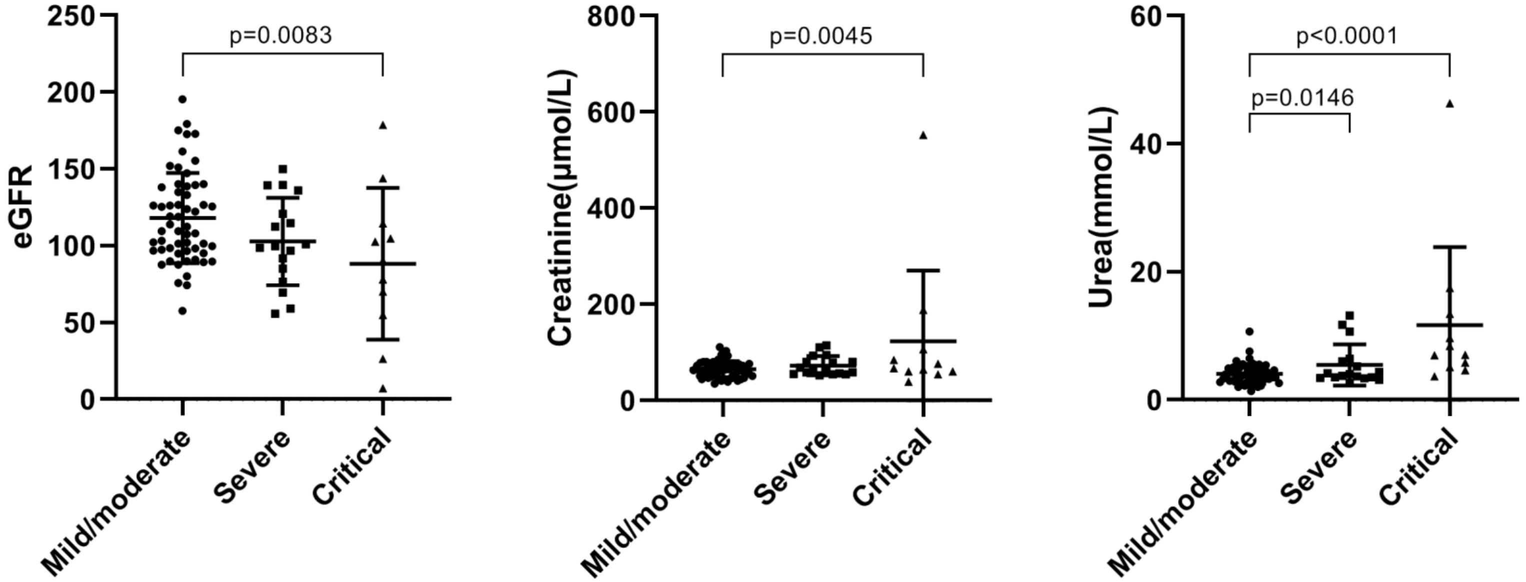

Data are presented as mean values ± SD. n(mild/moderate)=57,n(severe)=17,n(critical)=11. p values of three groups are from ordinary one-way ANOVA. p(eGFR)=0.0125, p(Creatinine)=0.0072, p(Urea)<0.0001. p values of each two groups which are displayed in figures are from two-side unpaired t test. eGFR = estimated glomerular filtration rate, Urea = serum urea nitrogen. Source data are provided as a Source Data file.

Supplementary Figure 2 SARS-CoV-2 viral mRNA are absent in the kidney tissues from HBV-MN patients.

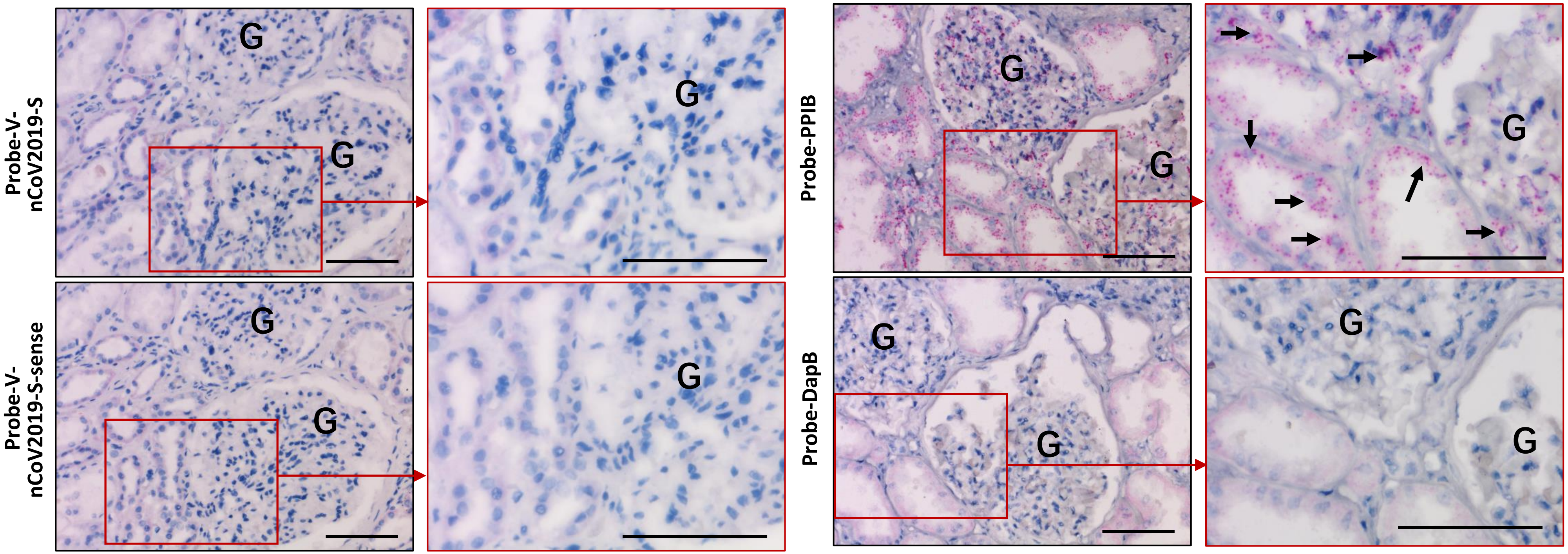

RNA in situ hybridization (ISH) in kidney tissues from hepatitis B virus-associated membranous nephropathy （HBV-MN） patients showed no presence of SARS-CoV-2 viral RNA. The housekeeping gene peptidylprolyl isomerase B (PPIB) are chosen as positive control probe, and bacterial gene diaminopimelate B (DapB) as negative control probe. G: glomerular. Scale bars = 100 μm. Data represent one of three technical replication each.

Supplementary Figure 3 Both SARS-NP and SARS-S antigens were absent in kidney tissues from trauma victims and HBV-MN patients.

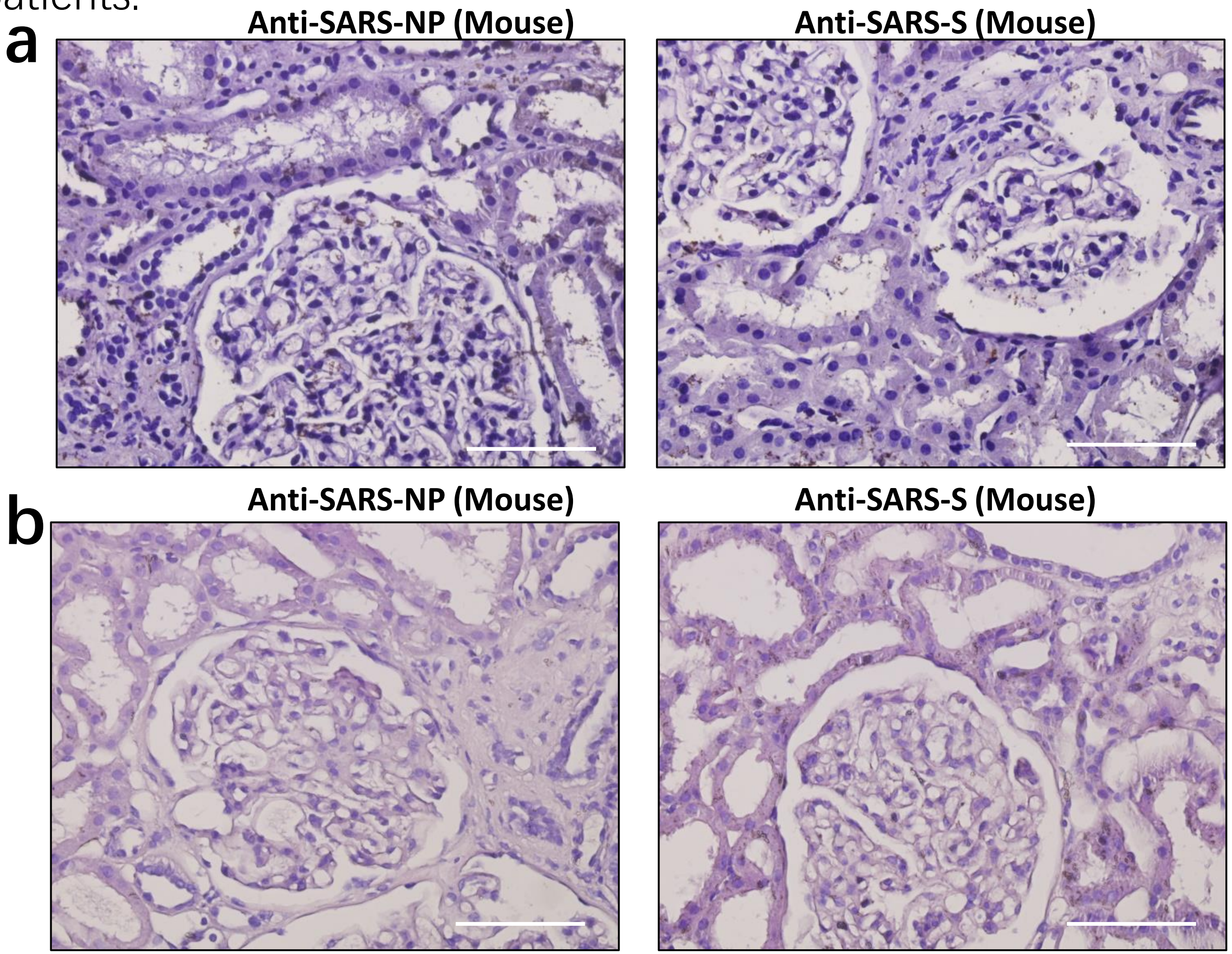

The kidney tissues from (**a**) trauma victim, and (**b**) hepatitis B virus-associated membranous nephropathy(HBV-MN) were incubated with anti-SARS-NP (nucleocapsid protein, mouse) or anti-SARS-S (spike, mouse) antibodies, and reactivity was confirmed by Immunohistochemistry. Scale bars= 100μm. Data represent one of three technical replication each.

Supplementary Figure 4 Validation of Anti-SARS-NP (nucleocapsid protein, Rabbit) antibodies.

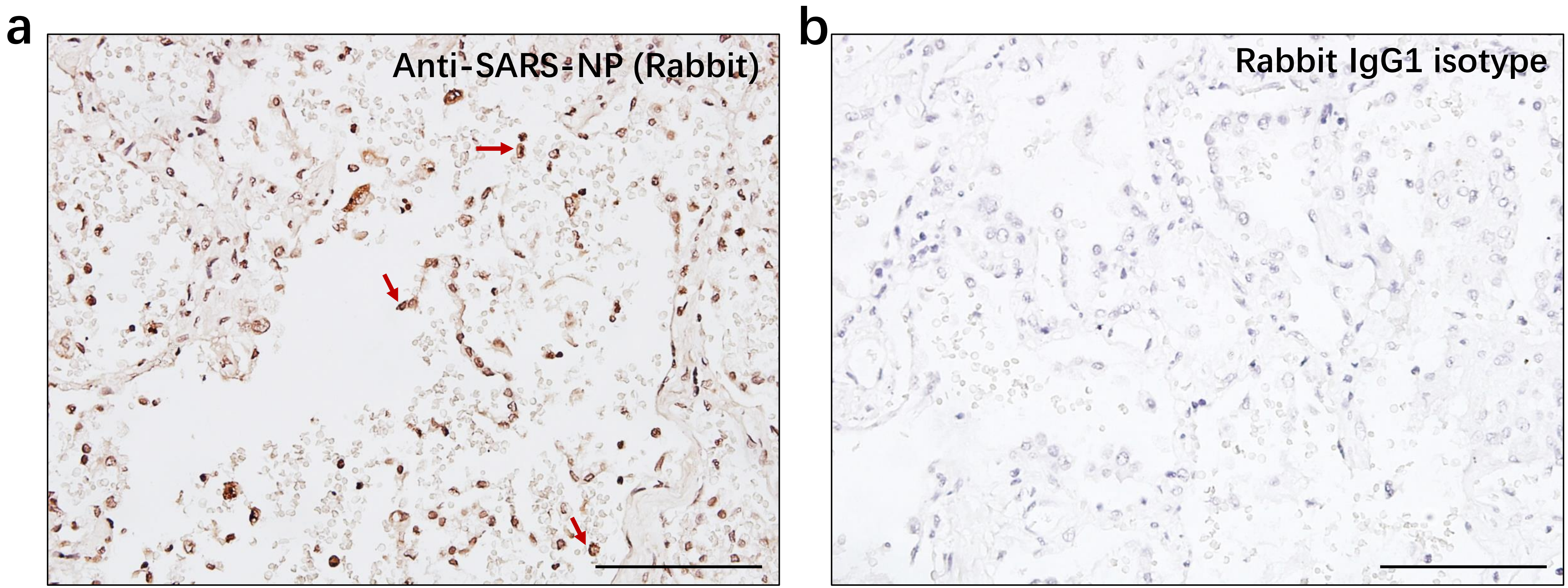

Lung tissues of COVID-19 patients. (**a**) positive control; (**b**) Rabbit IgG1 isotype control. Scale bars= 100μm. Data represent one of three technical replication each.

Supplementary Table1. Laboratory findings of the study patients

Group	AKI (N=23)	Non-AKI (N=62)	p value	Discovery
**Serum**				
Creatinine	84(67-104.5)	60.5(51-72)	0.0004	*
eGFR	77.96(64.29-89.55)	121.515(102.27-139.48)	< 0.0001	*
serum urea nitrogen	6.05(4.47-10.67)	3.715(3.255-4.665)	< 0.0001	*
Total bilirubin	13.6(9.35-18.85)	9.5(7.6-12.325)	0.0017	*
Direct bilirubin	3.8(2.7-6.25)	2.6(2.025-3.575)	0.0006	*
ALT	25(18.5-36)	22(13.25-30)	0.0502	
AST	32(25-58)	27.5(21-37.75)	0.0502	
Total protein	66.3(62.3-70.2)	64.4(61.675-70.05)	0.7079	
Albumin	36.4(31.35-38.7)	39.65(36.75-42.525)	0.0013	*
Globulin	28.2(24.8-34.65)	25.95(23.65-27.575)	0.0011	*
albumin/globulin ratio	1.24(0.93-1.565)	1.555(1.3725-1.65)	0.0005	*
Cholinesterase	6009(4731-7164.5)	7053(6247-7846.25)	0.0463	*
Prealbumin	0.15(0.075-0.2)	0.13(0.08-0.21)	0.9755	
Uric acid	313.5(230.5-390)	239(200.5-269.75)	0.0004	*
Carbon dioxide	22.4(21.175-25.025)	23.2(22.125-24.275)	0.3753	
Lactate (serum)	1.22(0.955-1.565)	1.05(0.9575-1.2275)	0.0187	*
Phosphorus	1.04(0.9-1.1475)	1.14(1-1.325)	0.0304	*
Magnesium	0.74(0.6275-0.795)	0.72(0.67-0.76)	0.0588	
Potassium	3.98(3.695-4.305)	3.74(3.4725-4.0575)	0.0885	
Sodium	138.2(136.25-139.95)	139.5(137.4-141)	0.0400	*
Chlorid	102.1(97.3-104.45)	102.8(100.125-105.325)	0.0712	
Calcium	2.14(2.045-2.215)	2.14(2.07-2.22)	0.5837	
creatine kinase	133(86-287)	99.5(80-144)	0.0033	*
CK-MB	18(15-30)	17(15-19)	0.7029	
lactate dehydrogenase	272(200-384)	209(169.25-254.5)	0.0055	*
HBDH	227(173-359)	172(143-219.25)	0.0013	*
CIP/CIAP	75(64-85)	68(53-77)	0.0593	
ESR	42(22.5-78.5)	35(17.75-68)	0.6154	
C-reactive protein	19.815(15.005-48.235)	14.77(6.1-34.725)	0.1835	
Blood glucose	5.71(5.195-6.245)	5.765(5.17-6.45)	0.6767	
Total cholestero	3.9(3.35-4.7575)	3.8(3.19-4.45)	0.2407	
Triglycerides	1.315(1.0375-1.515)	0.905(0.74-1.365)	0.2069	
high-density lipoprotein	1.075(0.9125-1.2175)	1.1(0.95-1.26)	0.5303	
low-density lipoprotein	2.26(1.8475-2.63)	2.065(1.7175-2.45)	0.2806	
Lipoprotein (a)	133(77.25-231)	77.5(32-178.25)	0.1522	
Free fatty acid	0.72(0.63-0.85)	0.595(0.455-0.705)	0.0491	*
**Urine**				
Specific density(SG)	1.02(1.013-1.029)	1.018(1.01-1.0235)	0.2262	
Urine pH	6(5.5-6.5)	6(6-6.5)	0.7659	
Leukocytes	2(0.6-9)	2.6(1.825-3.575)	0.1611	
Red blood cells	6.75(3.125-10.4)	8.35(3.85-13.975)	0.9422	
Epithelial	3.6(2.45-4.3)	7.9(4.625-14.325)	0.0837	
Bacteria	2(0-11)	4(0.75-13)	0.8459	
Abnormal color	2/17	3/51	0.4208	

turbid	4/17	7/51	0.3417	
Urobilinogen(URO)	0/17	0/51	1.0000	
Urine Bilirubin(BIL)	0/17	0/51	1.0000	
Uroketone body(KET)	6/17	12/51	0.3409	
Occult blood(BLD)	4/17	16/51	0.5387	
Urine protein(PRO)	7/17	13/51	0.2190	
Urine nitrite(NIT)	1/17	1/51	0.4072	
Urinary leukocyte	5/17	5/51	0.0481	*
Urine glucose(GLU)	7/17	6/51	0.0076	*

Data are median(IQR) or n/N. p values(two side) are from $\chi2$ or unpaired t test.
yr= years.AKI=acute kidney injury,eGFR=estimated glomerular filtration rate
ALT=alanine aminotransferase,AST=aspartate aminotransferase,
ESR=erythrocyte sedimentation rate,CK-MB=Creatine Kinase, MB Form
HBDH=Hydroxybutyrate dehydrogenase,CIP/CIAP=Calf intestinal alkaline phosphatase
Source data are provided as a Source Data file
