# Supplementary material for: Human kidney is a target for novel severe acute respiratory syndrome coronavirus 2 infection
Source: Nat Commun. 2021 May 4;12:2506. doi: 10.1038/s41467-021-22781-1 (PMC8096808; doi:10.1038/s41467-021-22781-1)
Supplement: Supplementary file 3 — Reporting Summary [file 41467_2021_22781_MOESM3_ESM.pdf]

nature research

Corresponding author(s): Yongwen Chen

Last updated by author(s): Feb 17, 2021

Reporting Summary

Nature Research wishes to improve the reproducibility of the work that we publish. This form provides structure for consistency and transparency in reporting. For further information on Nature Research policies, see Authors & Referees and the Editorial Policy Checklist.

Statistics

For all statistical analyses, confirm that the following items are present in the figure legend, table legend, main text, or Methods section.

n/a	Confirmed	
☐	☒	The exact sample size (*n*) for each experimental group/condition, given as a discrete number and unit of measurement
☐	☒	A statement on whether measurements were taken from distinct samples or whether the same sample was measured repeatedly
☐	☒	The statistical test(s) used AND whether they are one- or two-sided<br>*Only common tests should be described solely by name; describe more complex techniques in the Methods section.*
☐	☒	A description of all covariates tested
☐	☒	A description of any assumptions or corrections, such as tests of normality and adjustment for multiple comparisons
☐	☒	A full description of the statistical parameters including central tendency (e.g. means) or other basic estimates (e.g. regression coefficient) AND variation (e.g. standard deviation) or associated estimates of uncertainty (e.g. confidence intervals)
☐	☒	For null hypothesis testing, the test statistic (e.g. *F*, *t*, *r*) with confidence intervals, effect sizes, degrees of freedom and *P* value noted<br>*Give P values as exact values whenever suitable.*
☒	☐	For Bayesian analysis, information on the choice of priors and Markov chain Monte Carlo settings
☒	☐	For hierarchical and complex designs, identification of the appropriate level for tests and full reporting of outcomes
☒	☐	Estimates of effect sizes (e.g. Cohen's *d*, Pearson's *r*), indicating how they were calculated

Our web collection on statistics for biologists contains articles on many of the points above.

Software and code

Policy information about availability of computer code

Data collection	Microsoft office 2010 Excel
Data analysis	GraphPad Prism version 8.0 (GraphPad Software, Inc., San Diego, CA, USA)

For manuscripts utilizing custom algorithms or software that are central to the research but not yet described in published literature, software must be made available to editors/reviewers. We strongly encourage code deposition in a community repository (e.g. GitHub). See the Nature Research guidelines for submitting code & software for further information.

Data

Policy information about availability of data

All manuscripts must include a data availability statement. This statement should provide the following information, where applicable:

- Accession codes, unique identifiers, or web links for publicly available datasets
- A list of figures that have associated raw data
- A description of any restrictions on data availability

The authors declare that all data supporting the findings of this study are available within the article and its Supplementary Information Files or from the corresponding author upon request. Supplementary figure 1,Table1 and Supplementary table 1 have associated raw data. For protection of patients' privacy, only anonymized data are available. The source files are provided within attached files or from the corresponding authors by request.

Field-specific reporting

Please select the one below that is the best fit for your research. If you are not sure, read the appropriate sections before making your selection.

☒ Life sciences ☐ Behavioural & social sciences ☐ Ecological, evolutionary & environmental sciences

For a reference copy of the document with all sections, see nature.com/documents/nr-reporting-summary-flat.pdf

Life sciences study design

All studies must disclose on these points even when the disclosure is negative.

Sample size	Medical records from 85 COVID-19 patients (aged from 21 years to 92 years) with dynamic observation of renal function in General Hospital of Central Theatre Command in Wuhan from January 17, 2020 to March 3, 2020 were collected and retrospectively analyzed. The kidneys from six COVID-19 patients with postmortem examinations were collected, these perished COVID-19 patients were admitted in Jinyintan Hospital in Wuhan, China. The kidneys from two trauma victims' autopsies (male, 65 years old; female, 62 years old) and two biopsies from HBV- related glomerulonephritis (HBV-MN, male, 59 years; female 60 years, both patients were proteinuric, and both were classified as stage I-II) were also involved.
Data exclusions	There is no data exclusion.
Replication	This is a retrospective analysis. Moreover, in the experiments, all tests were repeated at least three times each.
Randomization	There are only one cohort. We tested all the samples available when the tissues are adequate, so the randomination are not needed.
Blinding	This is a retrospective analysis, and we collected all the involved patients' data, so the blinding is not needed.

Reporting for specific materials, systems and methods

We require information from authors about some types of materials, experimental systems and methods used in many studies. Here, indicate whether each material, system or method listed is relevant to your study. If you are not sure if a list item applies to your research, read the appropriate section before selecting a response.

Materials & experimental systems

n/a	Involved in the study	
☐	☒	Antibodies
☒	☐	Eukaryotic cell lines
☒	☐	Palaeontology
☒	☐	Animals and other organisms
☐	☒	Human research participants
☒	☐	Clinical data

Methods

n/a	Involved in the study	
☒	☐	ChIP-seq
☒	☐	Flow cytometry
☒	☐	MRI-based neuroimaging

Antibodies

Antibodies used	anti-SARS-CoV-2 nucleocapsid protein (NP) antibodies (clone ID: 019, rabbit IgG; Sino Biological, Beijing),anti-SARS-CoV-2 NP antibodies (ab273434, mouse monoclonal 6H3, Abcam), anti-SARS spike glycoprotein (S) antibodies (ab273433, mouse monoclonal 1A9, Abcam), anti-ACE2 (clone ID: 10108-RP01, rabbit IgG; Sino Biological), anti-CD8 (Clone ID:4B11, mouse IgG2b; BIO-RAD), anti-CD68 (Clone ID:KP1,mouse IgG1; BIO-RAD), anti-CD56 (Clone ID:123C3, mouse IgG1; BIO-RAD), anti-C5b-9 (clone ID: aE11, mouse IgG; Dakocytomation), anti-DP2 (sc-271898, mouse IgG1; Santa cruz Biotechnology) or anti-PDS (ab182141, rabbit IgG1; Abcam),rabbit-isotype antibody controls (Dako), Goat anti-Rabbit IgG (H+L) secondary antibody, HRP (#31460, Invitrogen), Goat anti-Mouse secondary antibody, HRP (PA1-74421, ThermoFisher),Alexa Fluor® 555-conjugated goat anti- rabbit IgG antibodies (Invitrogen, San Diego, CA, USA), Alexa Fluor® 488-conjugated goat anti- rabbit IgG1 antibodies (Invitrogen, San Diego, CA, USA)
Validation	The validation are proceeded by antibody producer or by negative or positive conrol.<br><br>anti-SARS-CoV-2 nucleocapsid protein (NP) antibodies (clone ID: 019, rabbit IgG; Sino Biological, Beijing): https://www.sinobiological.com/antibodies/cov-nucleocapsid-40143-r019 ;PubMed ID: 32292867<br><br>anti-SARS-CoV-2 NP antibodies (ab273434, mouse monoclonal 6H3, Abcam): https://www.abcam.cn/sars-nucleocapsid-protein-antibody-6h3-ab273434.html<br><br>anti-SARS spike glycoprotein (S) antibodies (ab273433, mouse monoclonal 1A9, Abcam): https://www.abcam.cn/sars-spike-glycoprotein-antibody-1a9-ab273433.html

anti-ACE2 (clone ID: 10108-RP01, rabbit IgG; Sino Biological): https://www.sinobiological.com/antibodies/human-ace2-10108-rp01; Koitka A, et al. (2008) Angiotensin converting enzyme 2 in the kidney. Clin Exp Pharmacol Physiol. 35(4): 420-5. Raizada MK, et al. (2007) ACE2: a new target for cardiovascular disease therapeutics. J Cardiovasc Pharmacol. 50(2): 112-9.

anti-CD8 (Clone ID:4B11, mouse IgG2b; BIO-RAD), https://www.bio-rad-antibodies.com/monoclonal/human-cd8-antibody-4b11-mca1817.html?f=s%2Fn

anti-CD68 (Clone ID:KP1,mouse IgG1; BIO-RAD), https://www.bio-rad-antibodies.com/monoclonal/human-cd68-antibody-kp1-mca5709.html?f=purified

anti-CD56 (Clone ID:123C3, mouse IgG1; BIO-RAD), https://www.bio-rad-antibodies.com/monoclonal/human-cd56-antibody-123c3-mca2693.html?f=purified

anti-C5b-9 (clone ID: aE11, mouse IgG; Dakocytomation), https://www.agilent.com/store/zh_CN/Prod-M077701-8/M077701-8

anti-DP2 (sc-271898, mouse IgG1; Santa cruz Biotechnology) https://www.scbt.com/zh/p/dp2-antibody-c-5?requestFrom=search

anti-PDS (ab182141, rabbit IgG1; Abcam)
https://www.abcam.cn/prostaglandin-d-synthase-lipocalinpds-antibody-ep12357-ab182141.html
rabbit-isotype antibody controls (Dako)
https://www.agilent.com/en/product/clinical-flow-cytometry/reagents-for-clinical-flow-cytometry/clinical-isotype-controls/rabbit-f-ab-2-992437

Goat anti-Rabbit IgG (H+L) secondary antibody, HRP (#31460, Invitrogen)
https://www.thermofisher.com/cn/zh/antibody/product/Goat-anti-Rabbit-IgG-H-L-Secondary-Antibody-Polyclonal/31460

Goat anti-Mouse secondary antibody, HRP (PA1-74421, ThermoFisher)
https://www.thermofisher.com/cn/zh/antibody/product/Goat-anti-Mouse-IgG1-Secondary-Antibody-Polyclonal/PA1-74421

Alexa Fluor® 555-conjugated goat anti- rabbit IgG antibodies (Invitrogen, San Diego, CA, USA)
https://www.thermofisher.com/cn/zh/antibody/product/Donkey-anti-Rabbit-IgG-H-L-Highly-Cross-Adsorbed-Secondary-Antibody-Polyclonal/A-31572

Alexa Fluor® 488-conjugated goat anti- rabbit IgG1 antibodies (Invitrogen, San Diego, CA, USA)
https://www.thermofisher.com/cn/zh/antibody/product/Donkey-anti-Rabbit-IgG-H-L-Highly-Cross-Adsorbed-Secondary-Antibody-Polyclonal/A-21206

Human research participants

Policy information about studies involving human research participants

Population characteristics

Medical records from 85 COVID-19 patients (37 female, aged from 21 years to 92 years) with dynamic observation of renal function in General Hospital of Central Theatre Command in Wuhan from January 17, 2020 to March 3, 2020 were collected and retrospectively analyzed. Postmortem autopsies were conducted on six patients who had been admitted in Jinyintan Hospital.The kidneys from two trauma victims' autopsies (male, 65 years old; female, 62 years old) and two biopsies from HBV-related glomerulonephritis (HBV-MN, male, 59 years; female 60 years, both patients were proteinuric, and both were classified as stage I-II) were also involved as control.

Recruitment

showed above.

Ethics oversight

This study was approved by the National Health Commission of China and Ethics Commission of General Hospital of Central Theatre Command ([2020]017-1) and Jinyintan Hospital (KY-2020-15.01). Due to the urgent need of treating this emerging epidemic, as well as the anonymization and retrospective analysis of clinical data, the written informed consent of the eighty-five patients with clinical data analyzed (shown in Table 1, Supplementary Table 1, Supplementary Figure 1) were waived by the Ethical Committee of General Hospital of Central Theatre Command. The written informed consent of patients whose post-mortem tissues were collected was signed by their family before the autopsies.

Note that full information on the approval of the study protocol must also be provided in the manuscript.
